# Supplementary material for: Call detail record aggregation methodology impacts infectious disease models informed by human mobility
Source: PLoS Comput Biol. 2023 Aug 10;19(8):e1011368. doi: 10.1371/journal.pcbi.1011368 (PMC10443843; doi:10.1371/journal.pcbi.1011368)
Supplement: S1 Text — Fig A in S1 Text. Comparison of individual origin-destination pairs between networks. The difference in the average daily volume of travel for the 5,804 individual origin-destination pairs common to both networks. Note that because of the difference between aggregation methodologies, many origin-destination pairs are censored in the Sequential network. Further, note that some pairs have higher volumes of travel in the Sequential network compared to the All Pairs network. This is caused by empirical differences in the volume of travel on specific days (certain origin-destination pairs in the Sequential network may have high counts on particular days but otherwise are censored). Fig B in S1 Text. The number of cell sites per district. The spatial distribution of cell sites, showing a high density of cell sites in urban areas. Base map data are publicly available under the MIT licence from: https://github.com/hamishgibbs/ghana_cdr_aggregation. Fig C in S1 Text. Number of cell sites by population. The number of cell sites compared to the population in individual districts. Fig D in S1 Text. Comparison of empirical and modelled travel networks. a) Empirical networks from each aggregation methodology. b) Movement networks modelled using the exponential gravity model. Distance kernels show the number of travellers by the distance of network connections in the c) empirical and d) modelled networks. Fig E in S1 Text. Comparison of empirical and modelled travel networks. a) Empirical networks from each aggregation methodology. b) Movement networks modelled using the power law gravity model. Distance kernels show the number of travellers by the distance of network connections in the c) empirical and d) modelled networks. Fig F in S1 Text. Comparison of empirical and modelled travel networks. a) Empirical networks from each aggregation methodology. b) Movement networks modelled using the radiation model. Distance kernels show the number of travellers by the distance of network [file pcbi.1011368.s001.docx]

**Supplementary Information**

**Call detail record aggregation methodology impacts infectious disease models informed by human mobility**

Hamish Gibbs*^1^, Anwar Musah^1^, Omar Seidu^2^, William Ampofo^3^, Franklin Asiedu-Bekoe^4^, Jonathan Gray^5^, Wole A. Adewole^5^, James Cheshire^1^, Michael Marks^6,7,8^, Rosalind M. Eggo^9^

^1^Department of Geography, University College London, London, United Kingdom.

^2^Ghana Statistical Service, Accra, Ghana.

^3^Noguchi Memorial Institute for Medical Research, University of Ghana, Legon, Accra, Ghana.

^4^Ghana Health Service, Ministry of Health, Accra, Ghana.

^5^Flowminder Foundation, Stockholm, Sweden.

^6^Department of Clinical Research, London School of Hygiene & Tropical Medicine, London, United Kingdom.

^7^Hospital for Tropical Diseases, University College London Hospital, London, United Kingdom.

^8^ Division of Infection and Immunity, University College London, London, United Kingdom.

^9^Department of Infectious Disease Epidemiology, London School of Hygiene & Tropical Medicine, London, United Kingdom.

***[*Hamish.Gibbs.21@ucl.ac.uk*](mailto:Hamish.Gibbs.21@ucl.ac.uk)


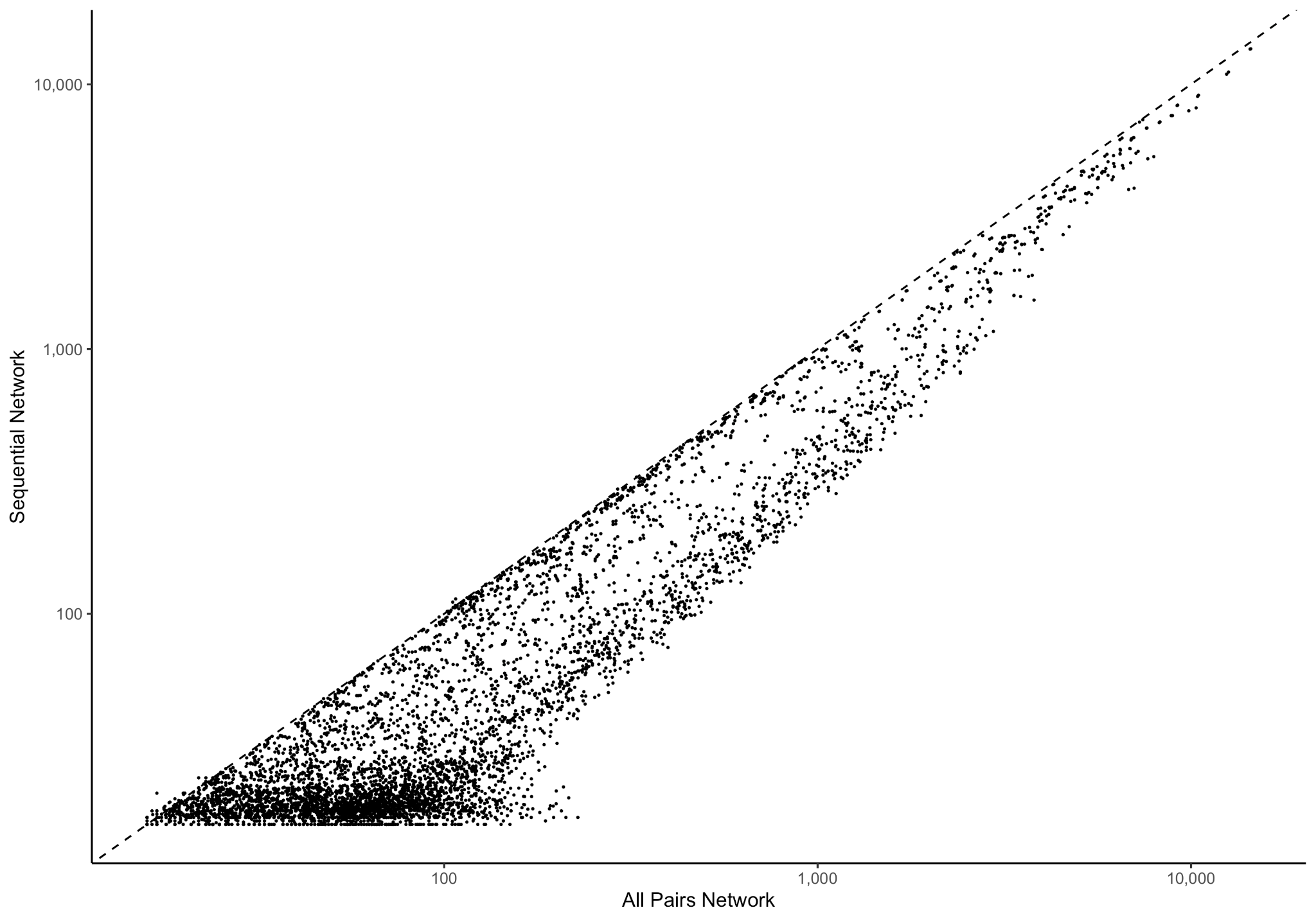


***Fig A. Comparison of individual origin-destination pairs between networks.*** *The difference in the average daily volume of travel for the 5,804 individual origin-destination pairs common to both networks. Note that because of the difference between aggregation methodologies, many origin-destination pairs are censored in the Sequential network. Further, note that some pairs have higher volumes of travel in the Sequential network compared to the All Pairs network. This is caused by empirical differences in the volume of travel on specific days (certain origin-destination pairs in the Sequential network may have high counts on particular days but otherwise are censored).*


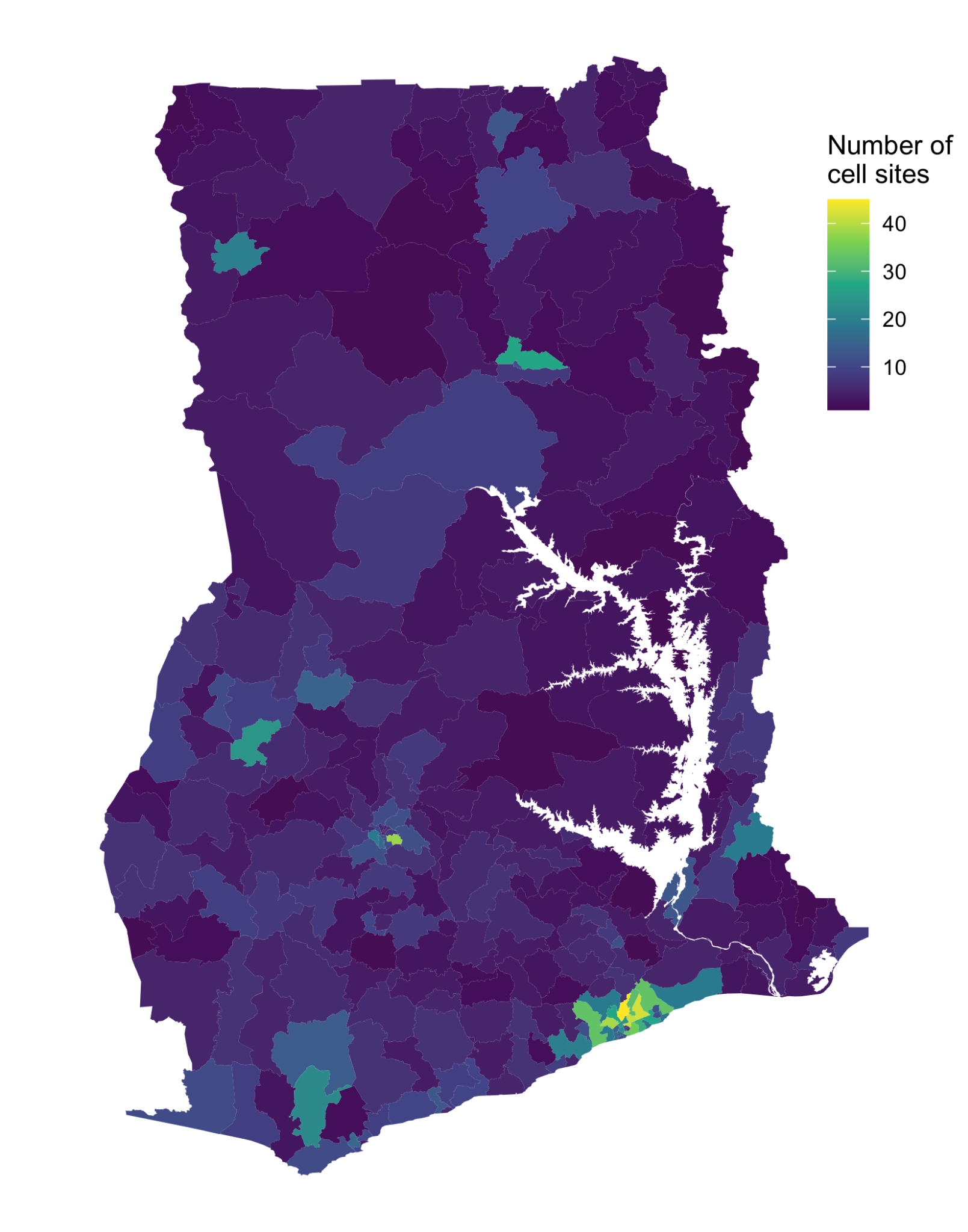


***Fig B. The number of cell sites per district.*** *The spatial distribution of cell sites, showing a high density of cell sites in urban areas. Base map data are publicly available under the MIT licence from:* [*https://github.com/hamishgibbs/ghana_cdr_aggregation*](https://github.com/hamishgibbs/ghana_cdr_aggregation)*.*


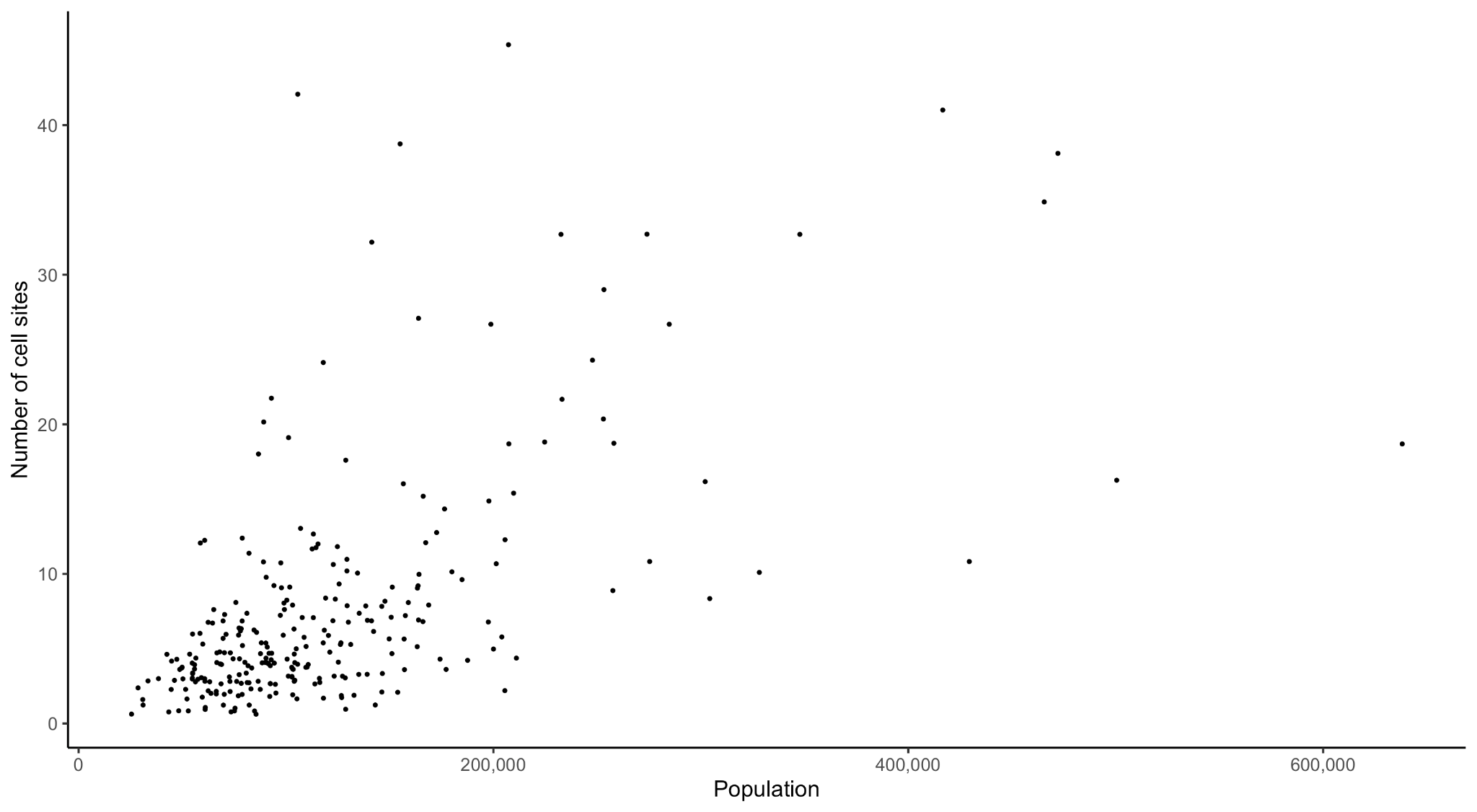


***Fig C. Number of cell sites by population.*** *The number of cell sites compared to the population in individual districts.*

*
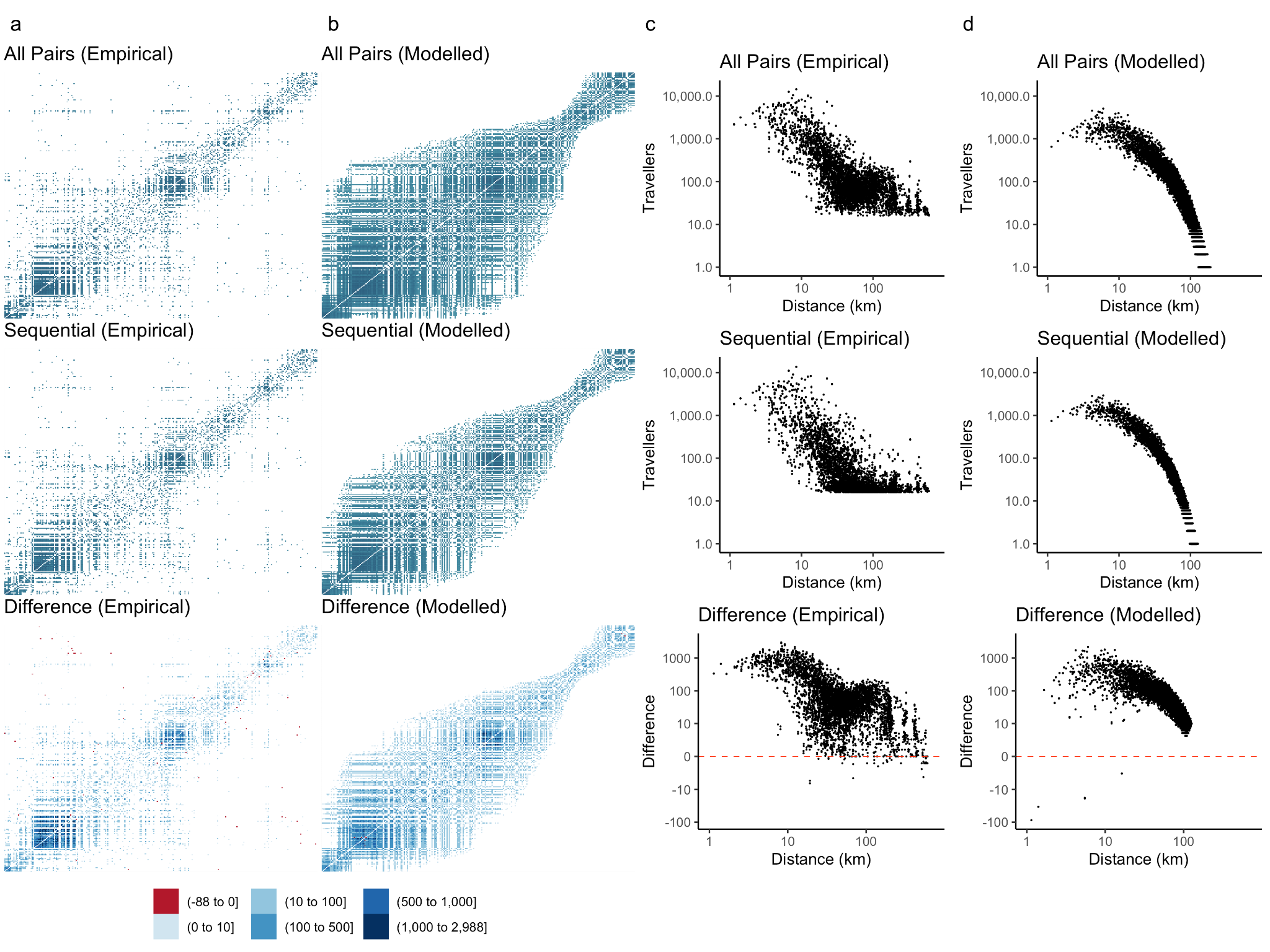
*

**Fig D*. Comparison of empirical and modelled travel networks.*** *a) Empirical networks from each aggregation methodology. b) Movement networks modelled using the exponential gravity model. Distance kernels show the number of travellers by the distance of network connections in the c) empirical and d) modelled networks.*

**
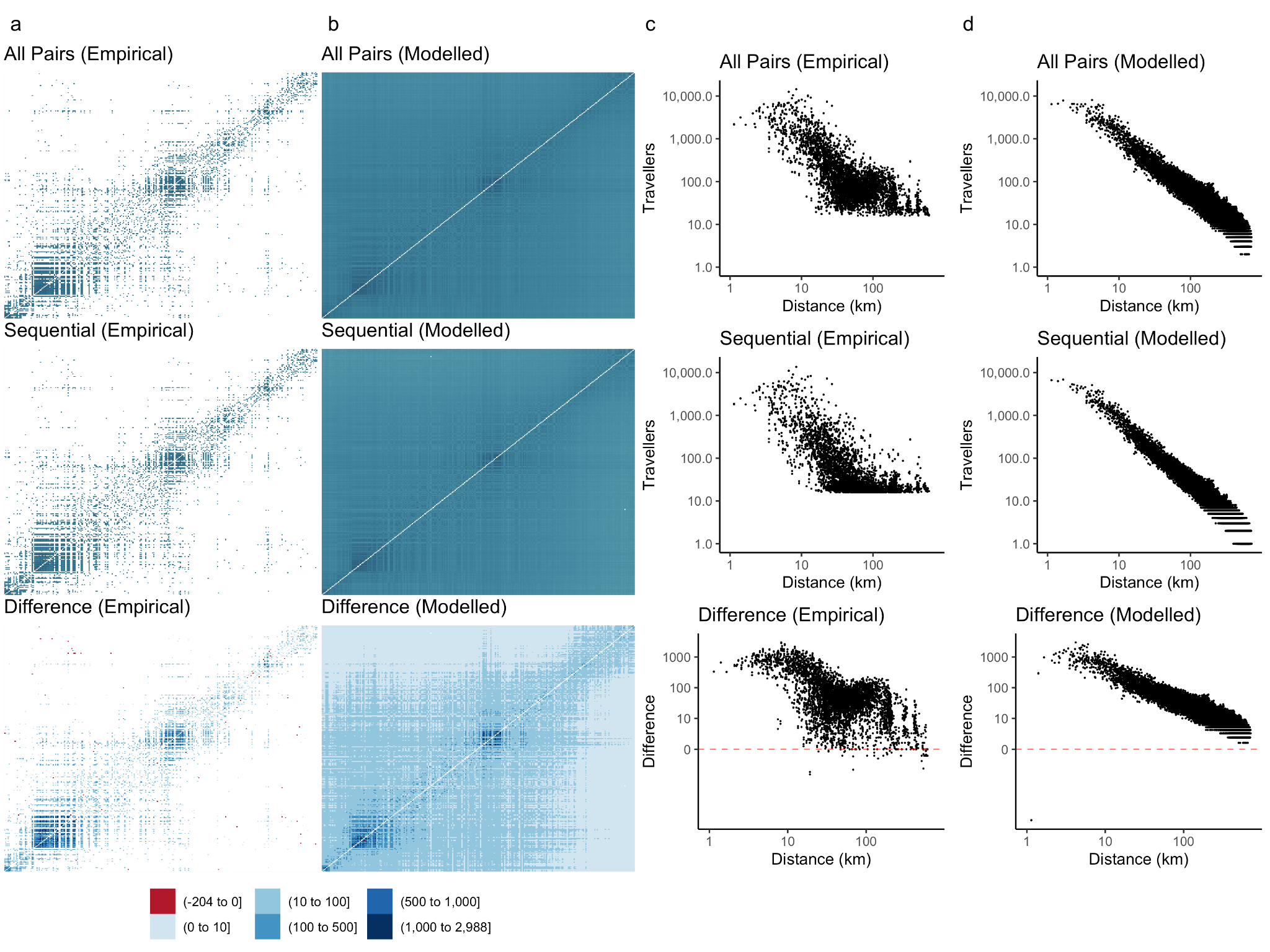
**

**Fig E*. Comparison of empirical and modelled travel networks.*** *a) Empirical networks from each aggregation methodology. b) Movement networks modelled using the power law gravity model. Distance kernels show the number of travellers by the distance of network connections in the c) empirical and d) modelled networks.*

*
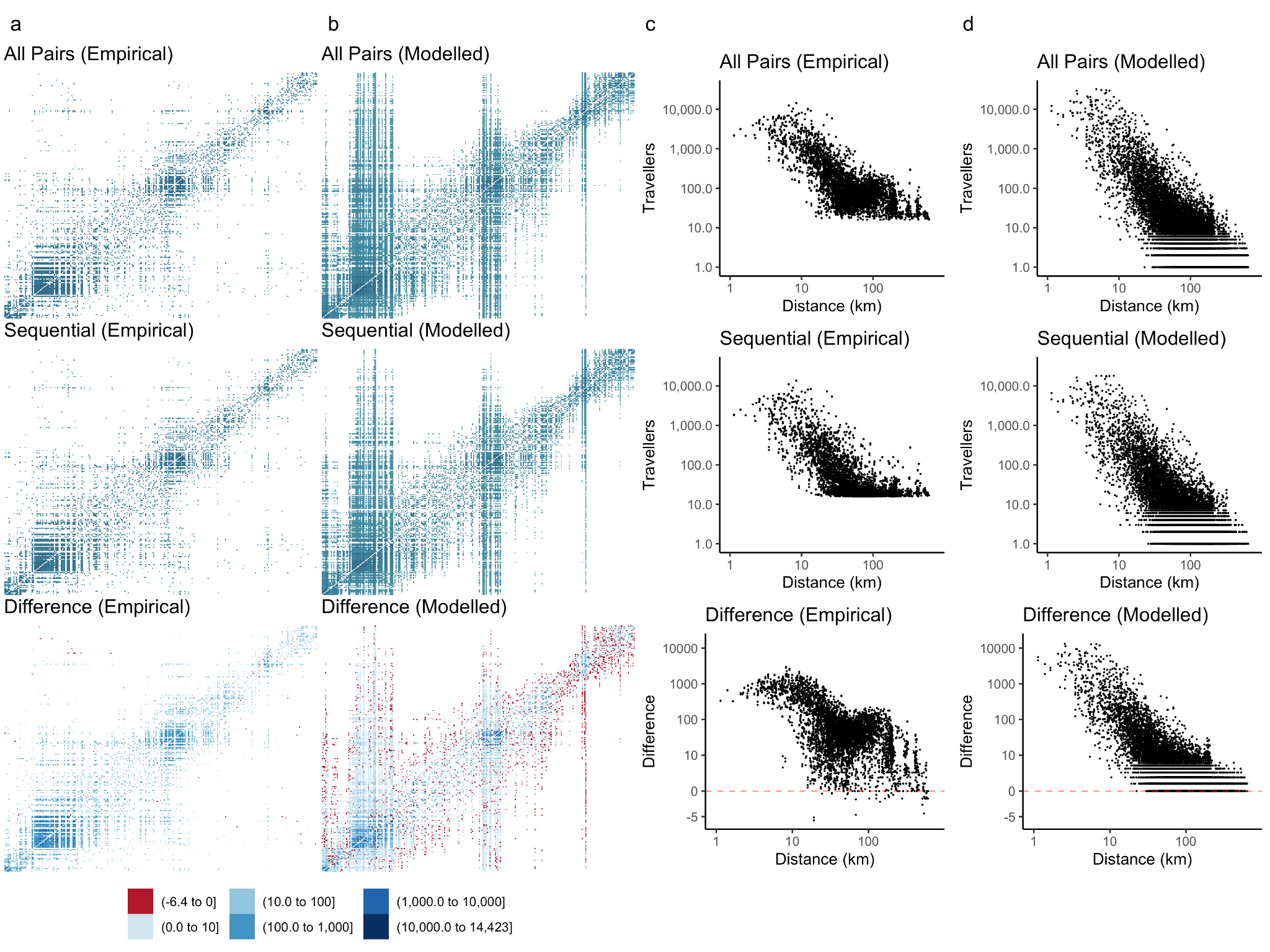
*

**Fig F*. Comparison of empirical and modelled travel networks.*** *a) Empirical networks from each aggregation methodology. b) Movement networks modelled using the radiation model. Distance kernels show the number of travellers by the distance of network connections in the c) empirical and d) modelled networks.*


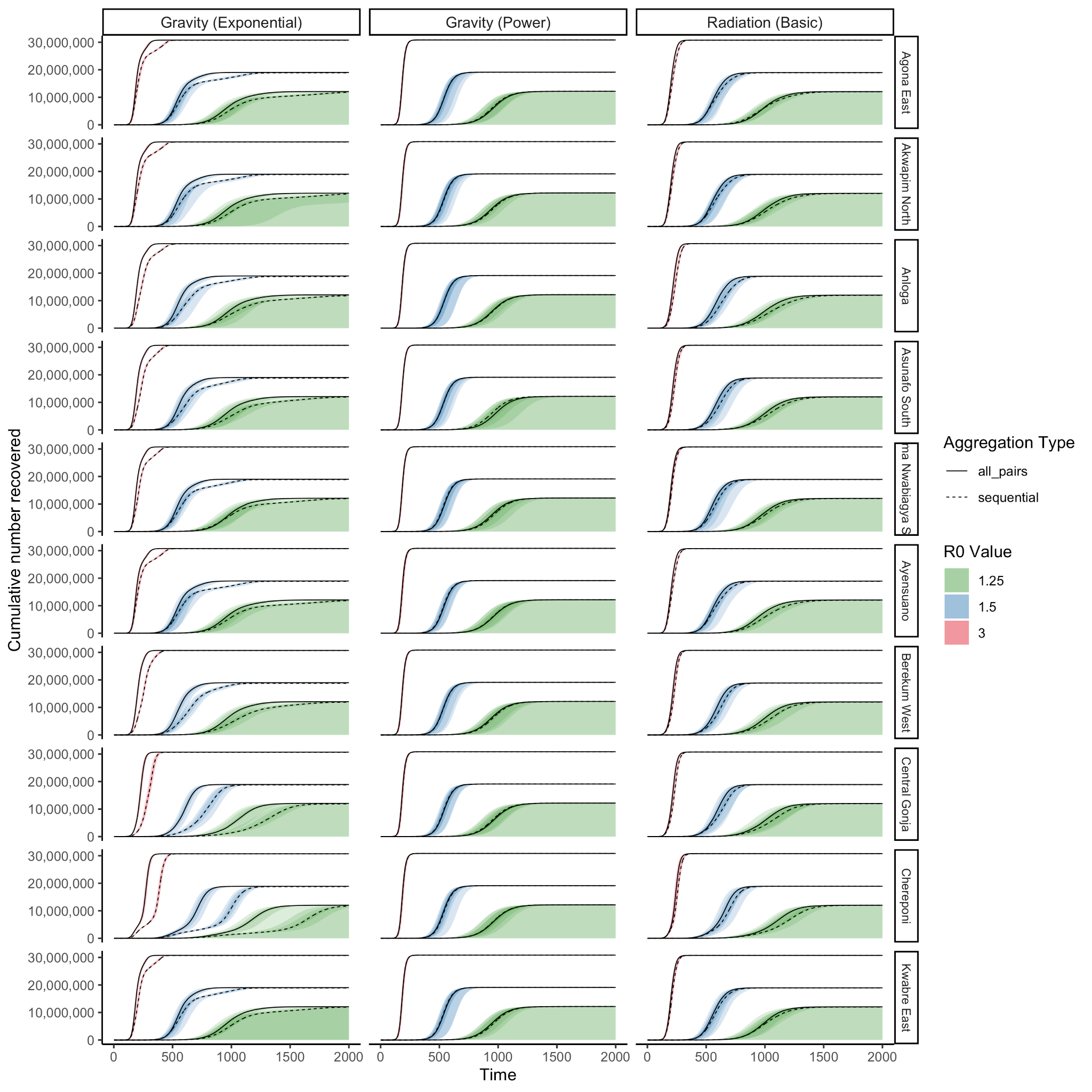


**Fig G*. Comparison of modelled national epidemics by aggregation methodology.*** *The difference in the number of individuals in the “recovered” compartment for a sample of 20 introduction locations, mobility models, and values of* R_0_*. Epidemics were modelled 100 times for each combination of aggregation methodology, introduction location,* R_0_*, and mobility model.*


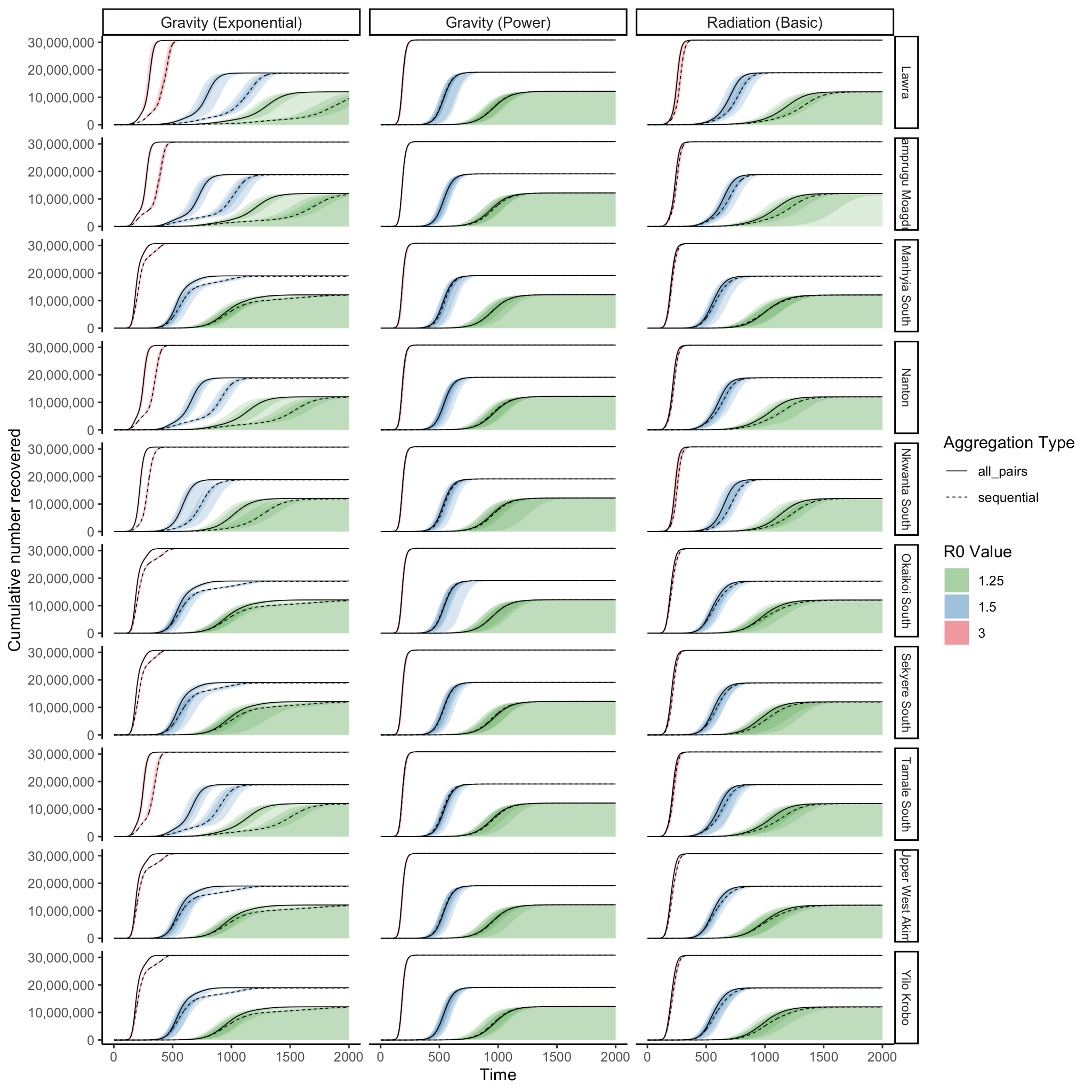


**Fig G*. Comparison of modelled national epidemics by aggregation methodology (continued).***

***
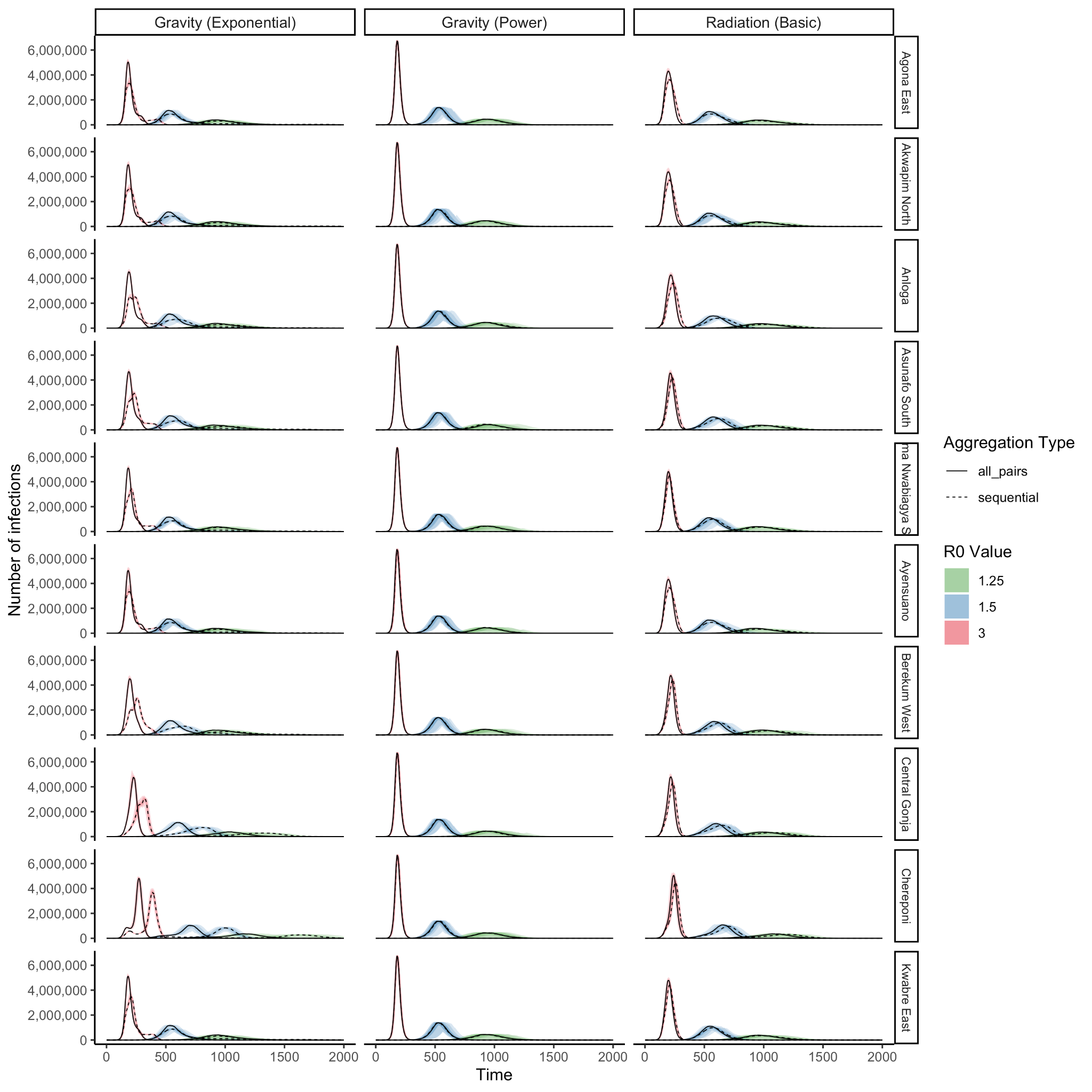
***

***Fig H. Comparison of modelled national epidemics by aggregation methodology.*** *The difference in the number of individuals in the “infected” compartment for a sample of 20 introduction locations, mobility models, and values of R_0_. Epidemics were modelled 100 times for each combination of aggregation methodology, introduction location, R_0_, and mobility model.*

***
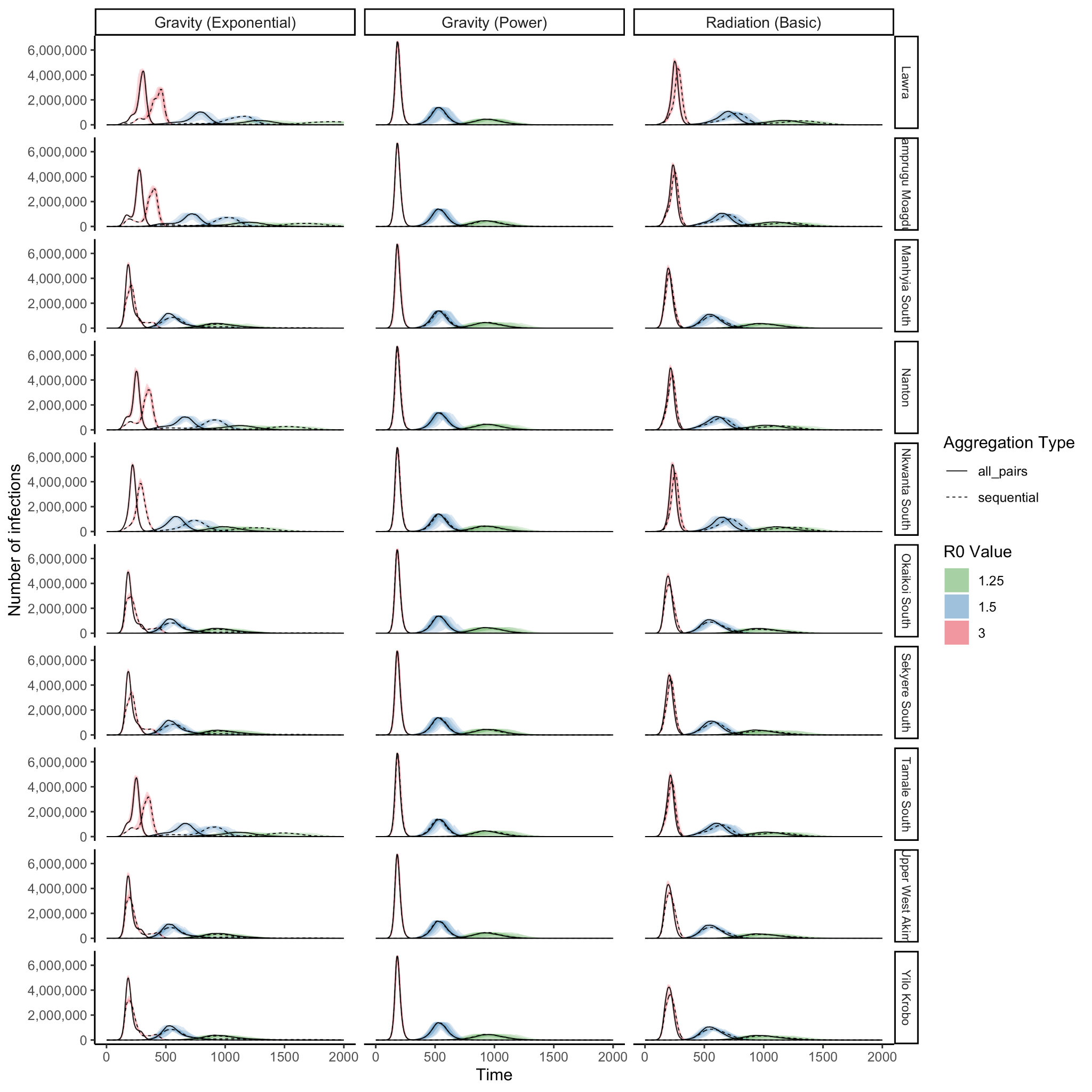
***

***Fig H. Comparison of modelled national epidemics by aggregation methodology (continued).***

|  |  | **Gravity (Exponential)** | **Gravity (Power)** | **Radiation (Basic)** | **Gravity (Exponential)** | **Gravity (Power)** | **Radiation (Basic)** |
| --- | --- | --- | --- | --- | --- | --- | --- |
| **Location** | **R_0_** | **Timing of 1^st^ infection** | | | **Timing of 5^th^ infection** | | |
| **Agona East** | **1.25** | 0.99 | 0.95 | 0.98 | 0.97 | 1 | 1 |
|  | **1.5** | 0.99 | 0.97 | 0.98 | 0.96 | 1 | 1 |
|  | **3** | 1 | 0.99 | 0.98 | 0.98 | 1 | 1 |
| **Akwapim North** | **1.25** | 0.99 | 0.94 | 0.98 | 0.97 | 0.99 | 1 |
|  | **1.5** | 0.99 | 0.98 | 0.98 | 0.97 | 1 | 1 |
|  | **3** | 1 | 0.99 | 0.99 | 0.97 | 1 | 1 |
| **Anloga** | **1.25** | 0.99 | 0.96 | 0.98 | 0.96 | 0.99 | 1 |
|  | **1.5** | 0.99 | 0.99 | 0.98 | 0.96 | 1 | 1 |
|  | **3** | 1 | 0.99 | 0.98 | 0.97 | 1 | 1 |
| **Asunafo South** | **1.25** | 0.97 | 0.93 | 0.96 | 0.96 | 0.98 | 0.99 |
|  | **1.5** | 0.98 | 0.98 | 0.96 | 0.96 | 0.99 | 0.99 |
|  | **3** | 0.99 | 0.99 | 0.96 | 0.97 | 0.99 | 0.99 |
| **Atwima Nwabiagya South** | **1.25** | 0.98 | 0.89 | 0.97 | 0.95 | 0.99 | 0.99 |
|  | **1.5** | 0.97 | 0.94 | 0.97 | 0.95 | 0.99 | 0.99 |
|  | **3** | 0.98 | 0.99 | 0.97 | 0.97 | 0.99 | 0.99 |
| **Ayensuano** | **1.25** | 0.99 | 0.95 | 0.98 | 0.97 | 0.99 | 0.99 |
|  | **1.5** | 0.99 | 0.97 | 0.98 | 0.96 | 1 | 1 |
|  | **3** | 0.99 | 0.99 | 0.99 | 0.98 | 1 | 1 |

***Table A. Spearman correlation coefficient comparing sequence infection under each aggregation method.*** *Correlation approaching one indicates high similarity between the sequence of infected districts under each aggregation methodology. All correlation coefficients are significant with p-value <0.0001.*

|  |  | **Gravity (Exponential)** | **Gravity (Power)** | **Radiation (Basic)** | **Gravity (Exponential)** | **Gravity (Power)** | **Radiation (Basic)** |
| --- | --- | --- | --- | --- | --- | --- | --- |
| **Location** | **R_0_** | **Timing of 1^st^ infection** | | | **Timing of 5^th^ infection** | | |
| **Berekum West** | **1.25** | 0.98 | 0.96 | 0.96 | 0.96 | 0.99 | 0.99 |
|  | **1.5** | 0.99 | 0.98 | 0.96 | 0.96 | 0.98 | 0.98 |
|  | **3** | 0.99 | 0.99 | 0.96 | 0.96 | 0.98 | 0.98 |
| **Central Gonja** | **1.25** | 0.97 | 0.95 | 0.91 | 0.96 | 0.96 | 0.98 |
|  | **1.5** | 0.97 | 0.98 | 0.93 | 0.95 | 0.97 | 0.98 |
|  | **3** | 0.99 | 0.99 | 0.9 | 0.94 | 0.98 | 0.99 |
| **Chereponi** | **1.25** | 0.96 | 0.94 | 0.89 | 0.94 | 0.96 | 0.97 |
|  | **1.5** | 0.97 | 0.97 | 0.92 | 0.95 | 0.96 | 0.97 |
|  | **3** | 0.99 | 0.99 | 0.91 | 0.96 | 0.96 | 0.96 |
| **Kwabre East** | **1.25** | 0.98 | 0.9 | 0.97 | 0.96 | 0.98 | 0.98 |
|  | **1.5** | 0.99 | 0.95 | 0.97 | 0.95 | 0.99 | 0.99 |
|  | **3** | 0.99 | 0.99 | 0.97 | 0.97 | 0.99 | 0.99 |
| **Lawra** | **1.25** | 0.98 | 0.98 | 0.88 | 0.96 | 0.94 | 0.96 |
|  | **1.5** | 0.99 | 0.99 | 0.91 | 0.95 | 0.96 | 0.96 |
|  | **3** | 0.99 | 0.99 | 0.91 | 0.95 | 0.94 | 0.94 |
| **Mamprugu Moagduri** | **1.25** | 0.96 | 0.95 | 0.86 | 0.95 | 0.98 | 0.98 |
|  | **1.5** | 0.98 | 0.98 | 0.92 | 0.95 | 0.98 | 0.99 |
|  | **3** | 0.99 | 0.99 | 0.93 | 0.97 | 0.97 | 0.98 |

***Table A. Spearman correlation coefficient comparing sequence infection under each aggregation method (continued).***

|  |  | **Gravity (Exponential)** | **Gravity (Power)** | **Radiation (Basic)** | **Gravity (Exponential)** | **Gravity (Power)** | **Radiation (Basic)** |
| --- | --- | --- | --- | --- | --- | --- | --- |
| **Location** | **R_0_** | **Timing of 1^st^ infection** | | | **Timing of 5^th^ infection** | | |
| **Manhyia South** | **1.25** | 0.97 | 0.88 | 0.97 | 0.96 | 0.97 | 0.97 |
|  | **1.5** | 0.98 | 0.96 | 0.97 | 0.97 | 0.99 | 0.99 |
|  | **3** | 0.98 | 0.99 | 0.97 | 0.97 | 0.99 | 0.99 |
| **Nanton** | **1.25** | 0.96 | 0.93 | 0.93 | 0.96 | 0.97 | 0.97 |
|  | **1.5** | 0.97 | 0.97 | 0.87 | 0.94 | 0.98 | 0.99 |
|  | **3** | 0.98 | 0.99 | 0.93 | 0.96 | 0.98 | 0.99 |
| **Nkwanta South** | **1.25** | 0.97 | 0.95 | 0.95 | 0.97 | 0.94 | 0.94 |
|  | **1.5** | 0.97 | 0.96 | 0.95 | 0.97 | 0.91 | 0.92 |
|  | **3** | 0.99 | 0.99 | 0.95 | 0.96 | 0.93 | 0.93 |
| **Okaikoi South** | **1.25** | 0.99 | 0.95 | 0.98 | 0.97 | 1 | 1 |
|  | **1.5** | 0.99 | 0.98 | 0.98 | 0.95 | 1 | 1 |
|  | **3** | 1 | 0.99 | 0.98 | 0.98 | 1 | 1 |
| **Sekyere South** | **1.25** | 0.97 | 0.89 | 0.96 | 0.96 | 0.99 | 0.99 |
|  | **1.5** | 0.98 | 0.95 | 0.97 | 0.95 | 0.98 | 0.98 |
|  | **3** | 0.98 | 0.99 | 0.98 | 0.96 | 0.99 | 0.99 |
| **Tamale South** | **1.25** | 0.97 | 0.95 | 0.92 | 0.96 | 0.97 | 0.99 |
|  | **1.5** | 0.98 | 0.97 | 0.9 | 0.95 | 0.98 | 0.99 |
|  | **3** | 0.99 | 0.99 | 0.9 | 0.96 | 0.98 | 0.99 |

***Table A. Spearman correlation coefficient comparing sequence infection under each aggregation method (continued).***

|  |  | **Gravity (Exponential)** | **Gravity (Power)** | **Radiation (Basic)** | **Gravity (Exponential)** | **Gravity (Power)** | **Radiation (Basic)** |
| --- | --- | --- | --- | --- | --- | --- | --- |
| **Location** | **R_0_** | **Timing of 1^st^ infection** | | | **Timing of 5^th^ infection** | | |
| **Upper West Akim** | **1.25** | 0.99 | 0.94 | 0.98 | 0.95 | 1 | 0.99 |
|  | **1.5** | 0.99 | 0.97 | 0.98 | 0.95 | 1 | 1 |
|  | **3** | 0.99 | 0.99 | 0.99 | 0.97 | 1 | 1 |
| **Yilo Krobo** | **1.25** | 0.99 | 0.95 | 0.97 | 0.94 | 1 | 1 |
|  | **1.5** | 0.99 | 0.97 | 0.98 | 0.96 | 1 | 1 |
|  | **3** | 0.99 | 0.99 | 0.98 | 0.98 | 1 | 1 |

***Table A. Spearman correlation coefficient comparing sequence infection under each aggregation method (continued).***


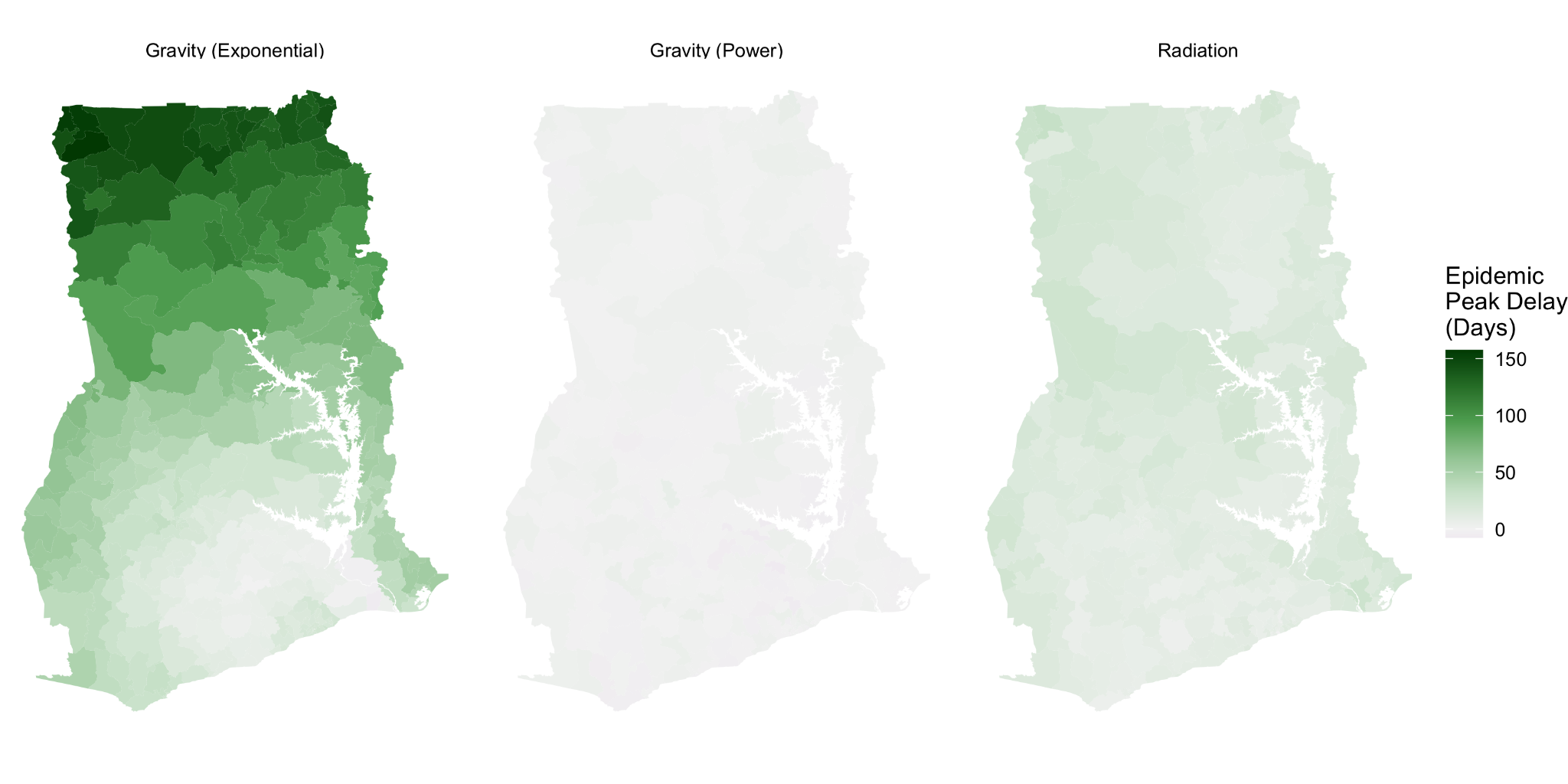


***Fig I. Influence of introduction location on the difference between aggregation methodologies.*** *Difference between the timing of the peak of a modelled epidemic with R_0_ = 3. Negative numbers indicate that the predicted epidemic based on the all pairs methodology was later than the epidemic predicted based on the sequential methodology. Base map data are publicly available under the MIT licence from:* [*https://github.com/hamishgibbs/ghana_cdr_aggregation*](https://github.com/hamishgibbs/ghana_cdr_aggregation)*.*


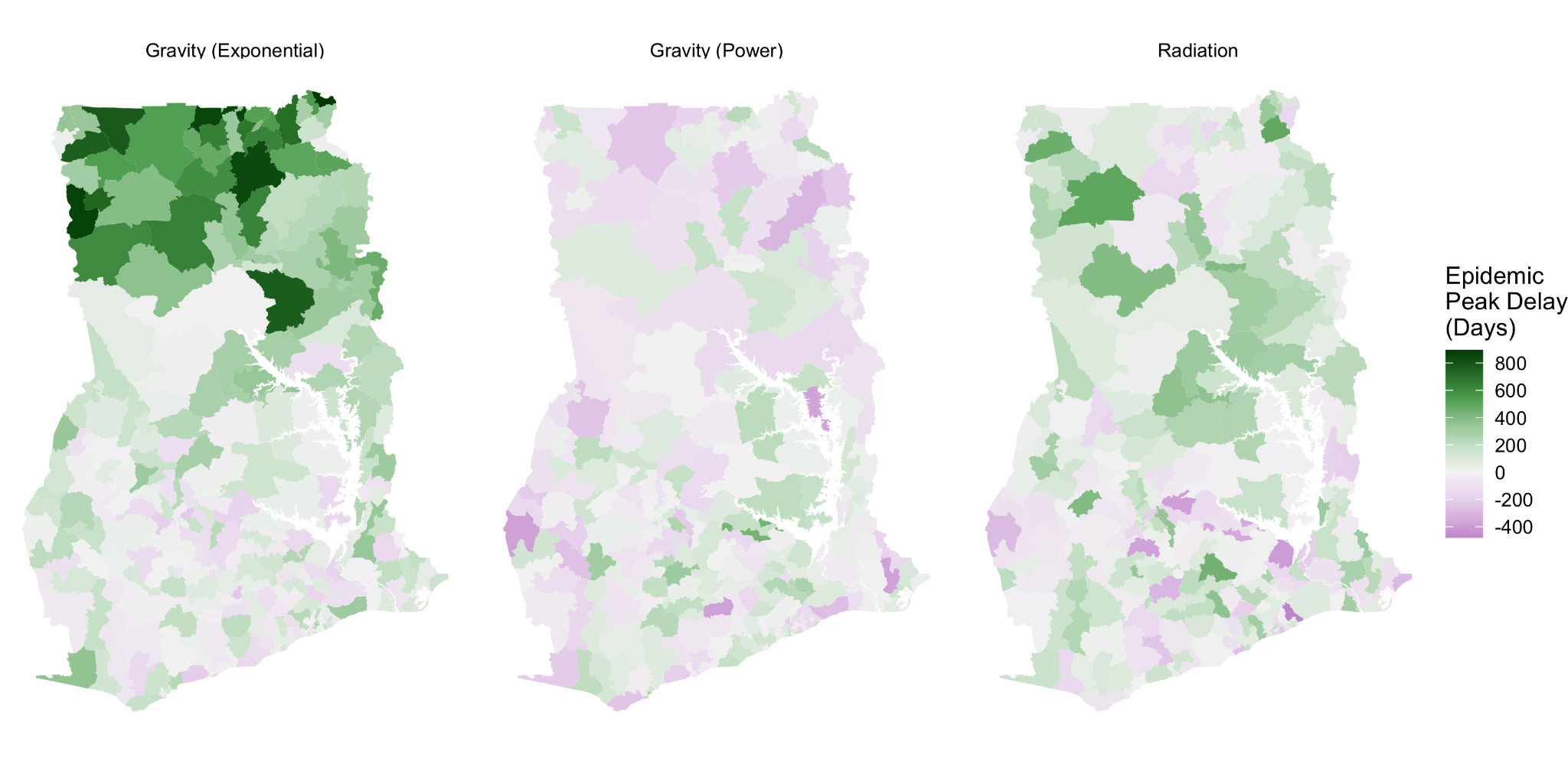


***Fig J. Influence of introduction location on the difference between aggregation methodologies.*** *Difference between the timing of the peak of a modelled epidemic with R_0_ = 1.25. Negative numbers indicate that the predicted epidemic based on the all pairs methodology was later than the epidemic predicted based on the sequential methodology. Base map data are publicly available under the MIT licence from:* [*https://github.com/hamishgibbs/ghana_cdr_aggregation*](https://github.com/hamishgibbs/ghana_cdr_aggregation)*.*
