# Supplementary material for: Call detail record aggregation methodology impacts infectious disease models informed by human mobility
Source: PLoS Comput Biol. 2023 Aug 10;19(8):e1011368. doi: 10.1371/journal.pcbi.1011368 (PMC10443843; doi:10.1371/journal.pcbi.1011368)
Supplement: Attachment — Submitted filename: response_to_reviewers.docx [file pcbi.1011368.s002.docx]

Dear Editor,

Thank you for sending these reviewer comments, which were helpful in improving the paper. We have addressed each comment and have made modifications to the text and the analysis in response. Below we have given further information on each of the changes.

Thank you for the opportunity to revise this manuscript for publication.

Yours sincerely,

Hamish Gibbs

**1. Responses to Reviewer #1**

**1.1 Comment #1**

**Reviewer:** The authors study the sensitivity of different aggregation methods of Call Detail Records (CDR), which can be used as a proxy for population mobility. They analyze differences in both the network structure and epidemic dynamics. Specifically, they compare two different aggregate methods: all-pairs and sequential. This paper has a very specific succinct goal, which the authors articulate well and succeed in reaching. As an epidemic modeler who could use these data/methods I think the paper could be strengthened with some additional analysis. I also have a couple questions about the initial conditions of the epidemic model that I did not fully understand from the methods section.

**Response:** We are grateful for this positive review of our work and have updated the manuscript to address the comments raised by the reviewer. Primarily, we have clarified the initial conditions of the model, have introduced a greater number of simulation replicates for the SEIR metapopulation model, and have increased the detail with which we analyse differences in the characteristics of the modelled epidemics under each aggregation methodology.

**1.2 Comment #2**

**Reviewer:** The main epidemiological metrics discussed are the shapes of the epidemic trajectories and peak magnitude/timing. I would also be interested to know whether there are other epidemiological quantities that could be informative such as the length of the epidemic or attack rates.

**Response:** We agree that further epidemiological metrics would add more information for readers. We have therefore introduced three changes to the manuscript to address this:

1. We have added total attack rate as a metric. We have updated Figure 5 in the main manuscript to include plots of the cumulative number infected during the epidemic. Because nearly all epidemics run to completion, we find that there are relatively small differences in the final size of the epidemic for each value of R_0_. However, there are differences in the attack rate over time which we have now visualised, showing differences in the total number of infected individuals at different stages of the epidemic.
2. We have also included a comparison of a greater number of epidemic characteristics between aggregation methodologies. Table 4 shows the difference in: the timing of the peak, the size of the peak, the overall length of the epidemic, the average time of the 1^st^ infection in all districts, and the average time of the 5^th^ infection in all districts. Because of computational limitations, we selected a sample of 20 districts for which we performed 100 epidemic simulations under each of the parameter combinations. We then compared the results of these simulations for each aggregation methodology using an independent samples t-test.
3. We have included an analysis of the ordered time of infection arrival for different introduction locations (suggested by Reviewer #2), where we explore whether aggregation methodology changes the topology of the transmission network between subpopulations or only changes the speed with which an epidemic spreads. Generally, we find the latter, showing little change in the order of infected districts between aggregation methodologies, despite significant differences in the characteristics of epidemics under each methodology.

These additions increase the amount of information available to readers to understand and assess the impact of the aggregation methodology on the epidemic simulations.

**1.3** **Comment #3**

**Reviewer:** On the network comparison side of the work. I would be interested to see how much edge variation that is. Currently, the paper reports the macroscopic differences in the total trips and how the average daily outbound tips change with the population and cell site count. But I would be interested in the micro-level variation. For example for each source-target location pair how do the number of travelers vary. A simple visualization is a scatter plot of each pair where the x-axis

**Response:** We thank the reviewer for this suggestion and have added a new supplemental figure (Supplemental Figure 1) comparing the volume of travel for origin-destination pairs common to both networks. One challenge in making this comparison is that the all pairs network has a larger overall number of connections than the sequential network (and in the sequential network, many connections are censored on at least some of the days of the study period).

This is discussed in the main text:

For individual origin-destination pairs common to both networks, the sequential network had an average of 43% less travel compared to the all pairs network (Supplemental Figure 1). Aside from a higher overall volume of travel, the all pairs network was also more connected than the sequential network, with 13,523 connections compared to 5,805, a 57% difference.

The new supplemental figure is as follows:

***Supplemental Figure 1. Comparison of individual origin-destination pairs between networks.*** *The difference in the average daily volume of travel for the 5,804 individual origin-destination pairs common to both networks. Note that because of the difference between aggregation methodologies, many origin-destination pairs are censored in the Sequential network. Further, note that some pairs have higher volumes of travel in the Sequential network compared to the All Pairs network. This is caused by empirical differences in the volume of travel on specific days (certain origin-destination pairs in the Sequential network may have high counts on particular days but otherwise are censored).*

**1.4 Comment #4**

**Reviewer:** When the difference in peak time is calculated is it only using 10 realizations? That doesn’t seem like enough statistics and I don’t think the process is described.

**Response:** Unfortunately, because of the number of parameter combinations and that the model is stochastic, we have encountered computational limitations in increasing the number of simulation replicates for all introduction locations. In order to increase the number of simulation replicates, we performed 100 simulations for a subset of 20 districts for all parameter combinations, and 10 simulations for all districts under each parameter combination. This is described in the methods section along with the initial conditions of the model (below).

**1.5 Major Comment #5**

**Reviewer:** I don’t think I understand how the initial conditions are set. In the methods section it says that there are 100 index infections introduced to all districts in Ghana, but I thought the analysis only studies 5 source districts. Does 100 represent the initial number of infections or the number of realizations? The section then reports that 10 introductions are sampled, but does that refer the 10 epidemic curves shown in figure 4? The last paragraph in the method section is not very clear in describing the initial conditions.

**Response:** We apologise for the confusion. Previously, we introduced 100 index infections into a single district for each simulation but we have decreased this to 10 index infections, which is a more realistic initial condition. In Figure 5, this is repeated for 100 replicates across a sample of 20 districts to assess the stability of the simulation, while in Figure 6 it is repeated for 10 replicates for all districts. We have clarified the initial conditions in the methodology:

We assess the sensitivity of the model to the location of infection introduction by introducing 10 index infections into one district for a series of model simulations with different parameter combinations and introduction locations. Because of computational limits, we selected a random sample of 15 districts in Ghana and included an additional 5 districts: Accra (Greater-Accra Region), Kumasi (Ashanti Region), Tamale (Northern Region), and two rural districts: Lawra (Upper West Region) and Nkwanta South (Oti Region) to provide a representative sample of the rural and urban gradient of districts in Ghana. For these districts, we performed 100 epidemic simulations for three values of $R_{0}$: 1.25, 1.5, 3.0, and for each movement model: Gravity (Exponential), Gravity (Power Law), and Radiation (Basic). For all other districts, we performed 10 epidemic simulations for each parameter combination. We chose these values of $R_{0}$ to simulate an infection similar to COVID-19, with $R_{0}$ between 1 and 3. Because $R_{0}$ is not a model input parameter, we vary the transmission rate *β*, given a constant recovery rate, *γ*.

**1.6 Comment #6**

**Reviewer:** Minor question: Does the number of infections in the epidemic trajectory figure refer to the prevalence or incidence of infections in the SEIR model (daily?)?

**Response:** This figure refers to the prevalence, i.e. the daily number of individuals in the “Infected” compartment. We have updated this figure in the main text (Figure 5) with plots of the cumulative number in the Removed compartment to provide an indication of the difference in attack rates over time. We have also updated the figures of the infected individuals to include a greater number of simulation replicates and moved these figures to the supplement (Supplemental Figures 7, 8, 9, 10)

The main text figure has been updated as follows:

***Figure 5. Comparison of recovered individuals in simulated national epidemics by aggregation method, movement model, and R_0_.*** *Density intervals show median, 20%, 60%, and 90% density distributions for 100 epidemic simulations for each parameter combination. Difference between solid and dotted lines indicates variation in the progression of national epidemics caused by aggregation methodology.* *Tamale South, Manhyia South and Okaikoi South are urban districts in the 3 largest cities of Ghana, and Nkwanta South and Lawra are rural districts.*

**2. Responses to Reviewer #2**

**2.1 Comment #1**

**Reviewer:** In this study, the authors compare two different approaches to generate origin-destination matrices from mobile phone derived location data. Since individual trajectories from call-detail-records (CDR) can be aggregated into OD matrices in different ways, it is important to compare the results of different procedures. Moreover, as OD matrices are often used to parameterize spatial epidemic models, different aggregation methods could lead to different epidemic simulation outcomes. Indeed, this is what the study shows with numerical simulations. Given the huge and ever-increasing interest in using mobile phone data to inform epidemic models, the study represents a useful addition to the literature, and it provides a good example of the impact of aggregation methodologies.I believe the results will be of interest to the readership of PLOS Computational Biology. On a less positive side, I have some suggestions that I hope will help improving the manuscript, before recommending it for acceptance.

**Response:** We thank the reviewer for their comments on this work. We have updated the manuscript according to these recommendations and this has enhanced the manuscript.

**2.2 Comment #2**

**Reviewer:** I think the manuscript would greatly benefit from the addition of an initial subsection, at the beginning of the Results, that provides more details on the dataset used and the aggregation methodology. Given that the focus of the paper are the aggregation methods, I think the description in the Methods lacks some important details and that the aggregation part should be introduced earlier in the manuscript. More specifically:

a. The acronym CDR is used to refer to the data but then the authors explain that this includes calls, text messages and data usage. Usually, in the literature, CDRs refers to calls only and I would clarify this point early in the manuscript to avoid confusion.

b. It is not very clear what type of mobility is inferred from mobile phone data. Is it recurrent or not? Do the authors know the home location of a user? Can they distinguish between users? What do the OD matrices represent in this case? The number of daily movements made by different users between provinces? The number of distinct users moving between provinces?

c. To help the reader understanding these points, a schematic figure would be very helpful. I suggest adding a figure 1 that better explains the concepts of Table 1, and specifically describes how movements of one device are aggregated over time and in space.

**Response:**

We have updated the methods section to provide greater detail about the nature of the information included in the CDR data used in the study. Neither CDR aggregation methodology provides estimates of home locations or recurrent patterns of travel and were designed to provide rapid measures of mobility in response to the COVID-19 pandemic. This means that both methodologies have some limitations because of their lack of detail about the travel characteristics of individual devices.

The methods section has been updated as follows:

The CDR data used in this study do not include information on the district of residence for mobile devices, based for example, on where a device tends to be located at night. Instead, the data describe the daily number of travellers between pairs of districts. Data are recorded daily, and the aggregated mobility networks are the sum of travellers recorded across all individuals based on the sequence of districts to which a device connected each day.

We have also added a new section to the results to explain the difference between the CDR aggregation methodologies. This section includes a diagram (Figure 1) indicating the difference in mobility networks produced for an individual travelling through a sequence of districts and how each methodology results in differing numbers of edges in the OD network.

We have also clarified the nature of the CDR data in our analysis: the data records all network transactions between a mobile device and the cellular network including calls, SMS messages, and cellular data usage. Sometimes this data is referred to as XDR data, but we have retained the name “CDR” for clarity. We have updated the new results section to make this distinction explicit.

The results section has been updated as follows:

In this paper, we used Call Detail Record (CDR) data from Vodafone Ghana, a mobile network operator which collects CDRs from subscribers to calculate billing charges. The data used in this study records all transactions between a mobile device and the cellular network including calls, text (SMS) messages, and data usage (this data is sometimes referred to as XDR data). The approximate location of a device can then be estimated using the location of the connected cell tower. “Movement” of devices is derived from the sequence of locations in which a mobile device connects to a cell tower. We use CDR data aggregated to the level of districts (Administrative Level 2).

In this analysis, we explore the impact of two different CDR aggregation methodologies: “all pairs” and “sequential” (14). Given the movement of one device through the same sequence of districts, these methodologies produce a different movement network, one representing all connections between transit districts and one recording only sequential connections (Figure 1a,b). In the all-pairs network, long distance connections are retained at the expense of over-estimation of the volume of travel in the network, as devices may be counted more than once. Alternatively, in the sequential network, the number of trips in the network accurately represents the quantity of devices in the movement network while omitting long-distance connections (Figure 1d). Further, the sequential network will maintain a constant relationship between transit locations and network connections (Figure 1c) while in the all pairs network, an increase in the number of transit locations will accelerate the number of network connections for each device (and thereby increase the overall density of the network).

This section of the results includes a new diagram describing the differences between CDR aggregation methodologies for an individual travelling through a series of districts:

***Figure 1. Differences between CDR aggregation methodologies.*** *Synthetic networks showing the transport network created by a single device moving through four districts, aggregated using a) the sequential methodology and b) the all pairs methodology. The number of network connections with increasing numbers of transit districts in c) the sequential network, and d) the all pairs network.*

**2.3 Comment #3**

**Reviewer:** Although the study is novel, there are several examples in the literature of similar studies where authors compared different mobility data sources, often mobile phone derived, and/or mobility models for epidemic purposes. The list would be long, I am only citing a few ones, but I think more references should be added to provide context. For instance:

- Perrotta D, et al. (2022) Comparing sources of mobility for modelling the epidemic spread of Zika virus in Colombia. PLoS Negl Trop Dis 16(7): e0010565.

- Oidtman, R.J., et al. Trade-offs between individual and ensemble forecasts of an emerging infectious disease. Nat Commun 12, 5379 (2021).

- Kraemer, M.U.G. et al. Utilizing general human movement models to predict the spread of emerging infectious diseases in resource poor settings. Sci Rep 9, 5151 (2019).

- Engebretsen, Solveig, et al. Time-aggregated mobile phone mobility data are sufficient for modelling influenza spread: the case of Bangladesh. Journal of the Royal Society Interface 17.167 (2020): 20190809.

**Response:** We thank the reviewer for highlighting these and other studies and we have updated and extended the introduction in an effort to provide more context regarding previous research in this area:

The choice of CDR aggregation methodology is typically an initial step in modelling disease transmission, and as such, there is little research concerning the impact of these apparently minor methodological choices on the predictions of epidemiological models. However, there has been significant research interest in how different choices of mobility data, or different mobility models can impact the progression of an epidemic. This has included the comparison of empirical and modelled movement networks (18,24), varying mobility model constructions (19,25,26), and varying sources of empirical mobility data (2,27,28). A key task for human mobility researchers is to understand areas of stability in epidemiological models informed by human mobility, where choices of mobility data or mobility model construction influence epidemiological models in predictable ways.

**2.4 Comment #4**

**Reviewer:** Similarly, there is some relevant literature about different methods to derive OD matrices from mobile phone data. This is a well-studied problem that deserves a more in-depth introduction for this study. First, it should be noted that “all-pairs” or “sequential” do not represent the only possible ways to derive OD matrices from mobile phone data. They are probably the most parsimonious ones, but more details could be used to derive commuting matrices. For instance, see:

- Bonnel, Patrick, Mariem Fekih, and Zbigniew Smoreda. "Origin-Destination estimation using mobile network probe data." Transportation Research Procedia 32 (2018): 69-81.

- Iqbal, Md Shahadat, et al. "Development of origin–destination matrices using mobile phone call data." Transportation Research Part C: Emerging Technologies 40 (2014): 63-74.

**Response:** We agree that this paper fits within a broader context of assessing the most accurate methods for representing human mobility using aggregated CDR data. We have added to the introduction to better address this area of research, including the references the reviewer highlights:

CDR data used in infectious disease research is typically produced as an aggregated, censored network describing the volume of travel between pairs of locations in a specified time period. The aggregation of CDR data transforms sensitive individual-level data into a description of population-level mobility, thereby reducing the risk of disclosing personally identifiable information. CDR aggregates can highlight different aspects of human mobility, from describing the volume of travel between sub-national districts (as addressed in this study)(14,15), to recurrent travel to/from a home district (16,17). CDR data may be limited by the size of the customer segment from which it is collected, or by the interaction between individuals and a mobile device. To address sparsity in the sample of individuals or travel behaviours captured in CDR data, the data may be rebalanced to better match official sources of travel data (18). Alternatively, CDR data may be used as the empirical input to models of human mobility which can estimate volumes of travel in sparse areas (19).

**2.5 Comment #5**

**Reviewer:** Finally, in the epidemiological analysis, it would be interesting to see, beyond the relative peak timing, whether the epidemic spreads between locations preserving the same order of infection or if this changes substantially. I am proposing something along the lines of what was done by Panigutti et al. (Royal Soc. Open Science 2017), by considering the ranking of locations by time of seeding (first infection recorded in a node). Although the epidemic may spread faster or slower on one network, the relative order of nodes that get infected may be preserved thus suggesting that the underlying network topology does not dramatically change.

**Response:** We have updated the manuscript to provide a more detailed analysis of the characteristics of the epidemic under different combinations of aggregation methodology, movement model construction, and R_0_ (also requested by Reviewer 1) The manuscript now provides an analysis of the length of the epidemic, the average timing of the 1st and 5th infections, as well as the timing and size of the epidemic peak.

Additionally, we felt that more detail regarding the change in arrival times produced under different aggregation methodologies would provide a helpful indication of whether the methodology change produces differences in the topology of the infection network, or only the speed with which the epidemic spreads along the mobility network. We have adopted part of the methodology of Panigutti et. al. (2017), analysing the Spearman rank correlation coefficient for the timing of 1st infection arrival in each district under different aggregation methodologies.

We have described this method in the methodology section:

To understand whether observed changes in epidemic characteristics are a result of increased “speed” of an epidemic, or are the result of changes in infection network topology, we calculate the Spearman rank correlation coefficient of the average arrival time of the 1st and 5th infections across 100 simulations. High correlation in the sequence of infections indicates greater similarity in the infection network, because of similarity in the sequence of infected districts between different aggregation methodologies.

And in the updated results section:

To understand whether differences in the progression of simulated epidemics were driven by differences in the topology of the transmission network, or merely indicated a “slowing” of similar infection trees under the sequential methodology, we calculated the Spearman’s rank correlation coefficient for the timing of epidemic arrival in each district. The Spearman rank correlation coefficient shows significant similarity in the sequences of infected districts under different aggregation methods, with a minimum correlation of 0.86 for all parameter combinations, with many simulated epidemics near or equal to 1 under either aggregation methodology (Supplemental Table 1). This indicates that, although epidemics may exhibit differences in the speed with which an infection spreads, these differences are largely driven by increases in the volume of travel around the movement network, not a change in the topology of the movement network, which would result in different sequences of infected districts.
